# Supplementary material for: Earthquake-enhanced dissolved carbon cycles in ultra-deep ocean sediments
Source: Nat Commun. 2023 Sep 11;14:5427. doi: 10.1038/s41467-023-41116-w (PMC10495447; doi:10.1038/s41467-023-41116-w)
Supplement: Supplementary file 1 — Supplementary Information [file 41467_2023_41116_MOESM1_ESM.pdf]

Supplementary Information for

Earthquake-enhanced dissolved carbon cycles in ultra-deep ocean sediments

Mengfan Chu[1], Rui Bao[1*], Michael Strasser[2], Ken Ikehara[3], Jez Everest[4], Lena Maeda[5], Katharina Hochmuth[6,7], Li Xu[8], Ann McNichol[9], Piero Bellanova[10], Troy Rasbury[11], Martin Koelling[12], Natascha Riedinger[13], Joel Johnson[14], Min Luo[15], Christian März[16,17], Susanne Straub[18], Kana Jitsuno[19], Morgane Brunet[20], Zhirong Cai[21], Antonio Cattaneo[22], Kanhsi Hsiung[23], Takashi Ishizawa[24], Takuya Itaki[3], Toshiya Kanamatsu[25], Myra Keep[26], Arata Kioka[27], Cecilia McHugh[28], Aaron Micallef[29], Dhananjai Pandey[30], Jean Noël Proust[20], Yasufumi Satoguchi[31], Derek Sawyer[32], Chloé Seibert[33], Maxwell Silver[34], Joonas Virtasalo[35], Yonghong Wang[36], Ting-Wei Wu[12,37], Sarah Zellers[38]

[1] Frontiers Science Center for Deep Ocean Multispheres and Earth System, and Key Laboratory of Marine Chemistry Theory and Technology, Ministry of Education, Ocean University of China; Qingdao, 266100, China.

[2] University of Innsbruck, Institute of Geology; Innsbruck, Austria.

[3] National Institute of Advanced Industrial Science and Technology (AIST), Geological Survey of Japan, Institute of Geology and Geoinformation; Ibaraki, 305-8567, Japan.

[4] British Geological Survey, Lyell Centre; Edinburgh EH14 4AP, UK.

[5] Center for Deep Earth Exploration, Japan Agency for Marine-Earth Science and Technology; Kanagawa, 236-0001, Japan.

[6] School of Geography, Geology and the Environment, University of Leicester, Leicester, UK.

[7] Australian Centre for Excellence in Antarctic Sciences, Institute for Marine and Antarctic Studies, University of Tasmania, 20 Castray Esplanade, Battery Point TAS 7004, Australia.

[8] NOSAMS Laboratory, Woods Hole Oceanographic Institution; Massachusetts, USA.

[9] Department of Geology and Geophysics, Woods Hole Oceanographic Institution; Massachusetts, USA.

[10] RWTH Aachen University, Institute of Neotectonics and Natural Hazards & Institute of Geology and Geochemistry of Petroleum and Coal; 52056 Aachen, Germany.

[11] Stony Brook University, Department of Geosciences; New York, 11794, USA.
[12] MARUM – Center for Marine Environmental Science, University of Bremen; Bremen, 28359, Germany.
[13] Boone Pickens School of Geology, Oklahoma State University; Oklahoma 74078, USA.
[14] University of New Hampshire, Department of Earth Sciences; New Hampshire, 03824, USA.
[15] Shanghai Engineering Research Center of Hadal Science and Technology, College of Marine Sciences, Shanghai Ocean University; Shanghai, China.
[16] School of Earth and Environment, University of Leeds; Leeds, LS2 9JT, UK.
[17] Institute for Geosciences, University of Bonn, Nussallee 8, 53115 Bonn, Germany.
[18] Lamont Doherty Earth Observatory, Geochemistry Division; New York, 10964, USA.
[19] Department of Life Science and Medical Bioscience, Waseda University; Tokyo 162-0041, Japan.
[20] Univ Rennes, CNRS, Géosciences Rennes, UMR 6118, 35000 Rennes, France.
[21] Kyoto University, Department of Geology and Mineralogy, Division of Earth and Planetary Sciences, Graduate School of Science; Kyoto, 606-8502, Japan.
[22] Geo-Ocean, UMR 6538, Univ Brest, CNRS, Ifremer; Plouzané, F-29280, France.
[23] Research Institute for Marine Geodynamics, JAMSTEC, Marine Geology and Geophysics Research Group, Subduction Dynamics Research Center; Kanagawa, 237-0061, Japan.
[24] International Research Institute of Disaster Science, Tohoku University, Sendai, 980-0845, Japan.
[25] Japan Agency for Marine-Earth Science and Technology (JAMSTEC), Research Institute of Marine Geodynamics (IMG); Yokosuka 237-0061, Japan.
[26] The University of Western Australia, Department School of Earth Sciences; Perth, Australia.
[27] Kyushu University, Department of Earth Resources Engineering; Fukuoka 819-0395, Japan.
[28] Queens College, City University of New York, School of Earth and Environmental Sciences; New York, 11367, USA.
[29] GEOMAR Helmholtz Centre for Ocean Research Kiel; Kiel D-24148, Germany.
[30] National Centre for Polar and Ocean Research, Ministry of Earth Sciences, Government of India; Goa 403 804, India.

[31] Lake Biwa Museum; Shiga 525-0001, Japan.
[32] The Ohio State University, School of Earth Sciences; Ohio, 43210, USA.
[33] Lamont Doherty Earth Observatory, Marine geology and geophysics division, New York, 10964, USA.
[34] Colorado School of Mines, Hydrologic Science and Engineering; Colorado, 80227, USA.
[35] Geological Survey of Finland (GTK); Espoo, 02151, Finland.
[36] Ocean University of China, Department of Marine Geosciences; Qingdao, 266100, China.
[37] Norwegian Geotechnical Institute, Oslo, Norway.
[38] University of Central Missouri, Department of Physical Sciences; Missouri 64093, USA.

*Corresponding author. Email: baorui@ouc.edu.cn

Supplementary Text 1. Sources of the dissolved carbon in the Japan Trench sediments.

The carbon isotopic signatures of interstitial water (IW) dissolved organic and inorganic carbon (DOC, DIC) in the Japan Trench are informative of their sources. The $\Delta^{14}C$ values of the near-surface IW at Sites M0081, M0083 and M0084 are significantly higher than the bottom water (BW) DOC in the Japan Trench and in the Mariana Trench[1] (Supplementary Fig. 1), thus excluding the BW as the major IW DOC source. On the other hand, the trench SOC is potentially composed of sinking particulate organic carbon (POC) and earthquake-introduced sedimentary organic carbon (SOC). The IW DOC in the Japan Trench is also clearly distinguished from the POC in the Japan Trench and the Mariana Trench[1,2] (Supplementary Fig. 1). The IW dissolved carbon is therefore mainly produced from the remineralization of earthquake-introduced SOC.

Earthquakes introduce significant amount of SOC with various provenances into the Japan Trench[3,4]. While both terrestrial[4] and marine[3] SOC are proposed to be dominant in turbidite deposits in the Japan Trench, the ^{13}C signatures of the DOC pool reveal its marine origin. Typical $\delta^{13}C$ values for the OC reservoirs include –25‰ for terrestrial biogenic OC, –20‰ for marine biogenic OC, 1‰ for biogenic carbonates and –29‰ for fossil OC[5]. The DOC in the Japan Trench is therefore mainly produced from the marine-sourced SOC, which can result from either selective degradation or dominance of marine-sourced OC in the SOC pool. In addition, the relatively low $\Delta^{14}C$ values (Supplementary Fig. 1) indicate that the source SOC pool is dominated by earthquake-remobilized pre-aged OC[4].

OC degradation and anaerobic oxidation of methane are two processes observed at the sulfate-methane transition zone (SMTZ)[6,7]. OC degradation typically produces ^{13}C-enriched DIC (~ –9‰), and anaerobic oxidation of methane produces ^{13}C-depleted DIC (~ –24‰)[7,8]. The ^{13}C signatures (–12.6‰ to –15.6‰, Fig. 2) of the DIC pools in the SMTZ and the variations of IW geochemistry parameters (Supplementary Fig. 3)

indicate that both OC degradation and methane diffusion exert controls on the DIC pool in the Japan Trench.

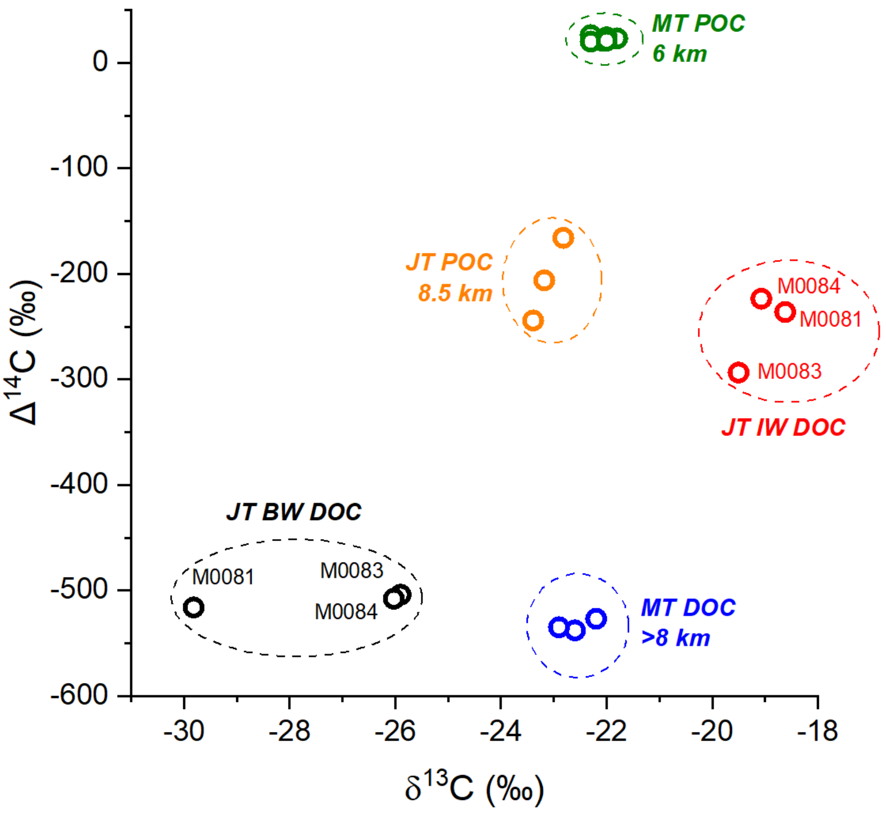

Supplementary Fig. 1. Comparisons of the carbon isotopic signatures between the BW DOC (black) and IW DOC (red) in the Japan Trench (JT, this study), the deep-water POC (orange) in the JT[2], and the deep-water DOC (blue) and POC (green) in the Mariana Trench (MT)[1].

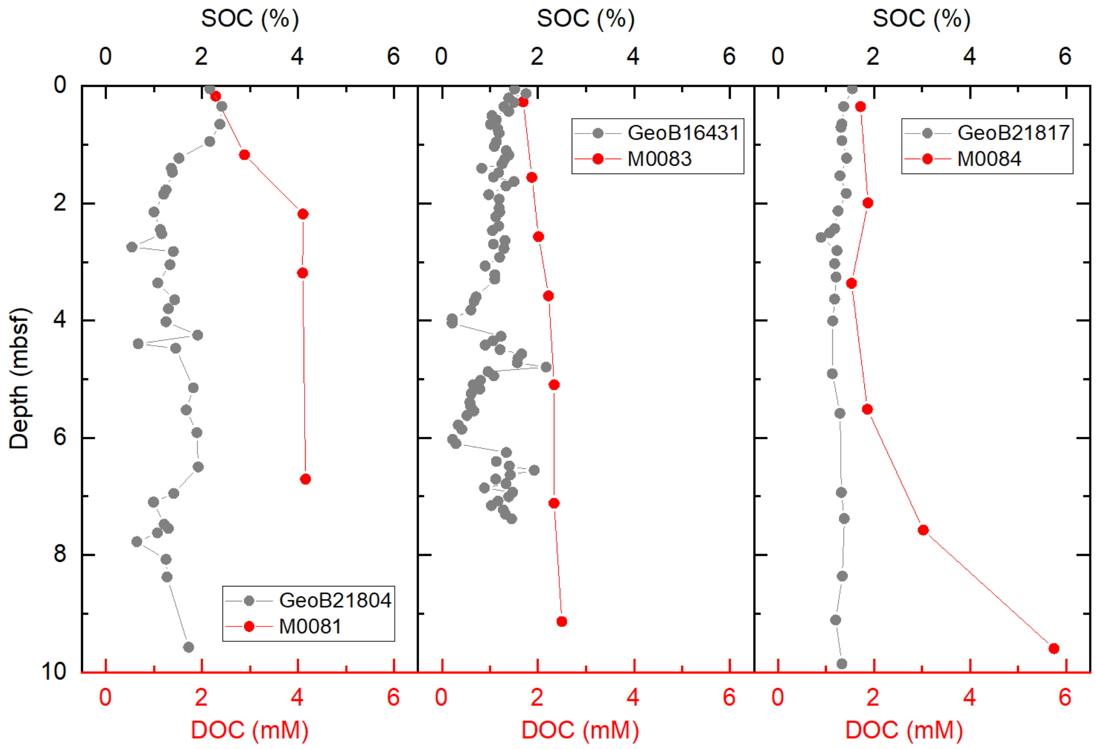

Supplementary Fig. 2. Comparisons of SOC and DOC concentrations between the upper 10 m of Sites M0081 (red) and GeoB21804 (grey) (located in the same southern basin), Sites M0083 (red) and GeoB16431 (grey), Sites M0084 (red) and GeoB21817 (grey) (located in the same northern basin). The different vertical variations between SOC and DOC concentrations imply that DOC production is not completely regulated by SOC content. The relatively short cores GeoB21804 and GeoB21817 were collected during R/V Sonne cruise SO251 in 2016, and core GeoB16431 was collected during R/V Sonne cruise SO219 in 2012. The GeoB core data are from refs.[3,4].

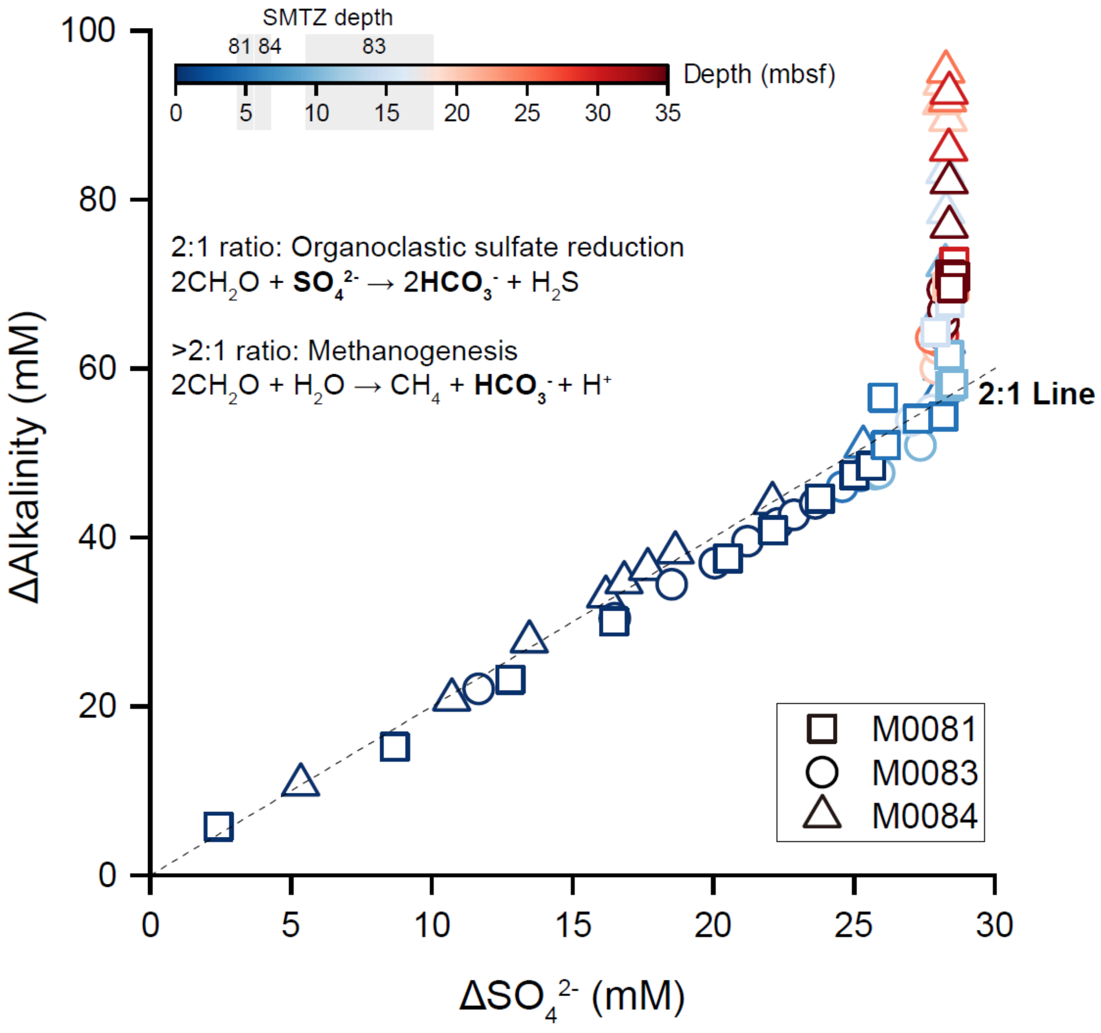

Supplementary Fig. 3. The differences of alkalinity and SO_4^{2-} concentrations between IW and BW (in absolute values, expressed as ΔAlkalinity and ΔSO_4^{2-}, respectively) at Sites M0081 (square), M0083 (circle) and M0084 (triangle). The SMTZ at each Site is illustrated by gray frames on the depth scale. The ratios of ΔAlkalinity to ΔSO_4^{2-} indicate influences caused by various biogeochemical processes on the IW. For instance, the >2:1 ratio indicates influence from methanogenesis, and the 2:1 ratio indicates influence from organoclastic sulfate reduction (i.e., OC remineralization) on the IW[9].

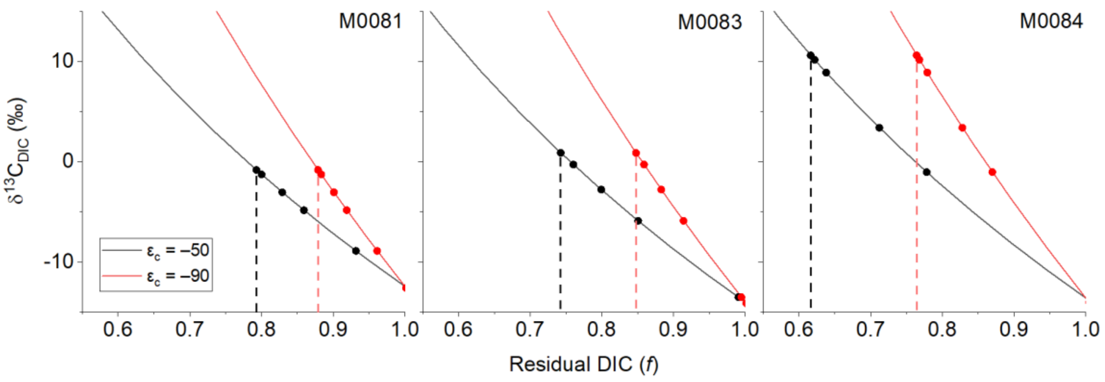

Supplementary Fig. 4. Relationships between $\delta^{13}C_{DIC}$ and the residual DIC (expressed as fraction, *f* ranges from 0 to 1) from methanogenesis. Both the upper and lower limits of isotope separation factor (ε_C) of microbial methanogenesis (–50‰, black; and –90‰, red)[10] are used. In the deepest IW at Sites M0081, M0083 and M0084, up to 38% (applying ε_C = –50‰) or 24% (applying ε_C = –90‰) of DIC is converted to methane (Supplementary Table 2).

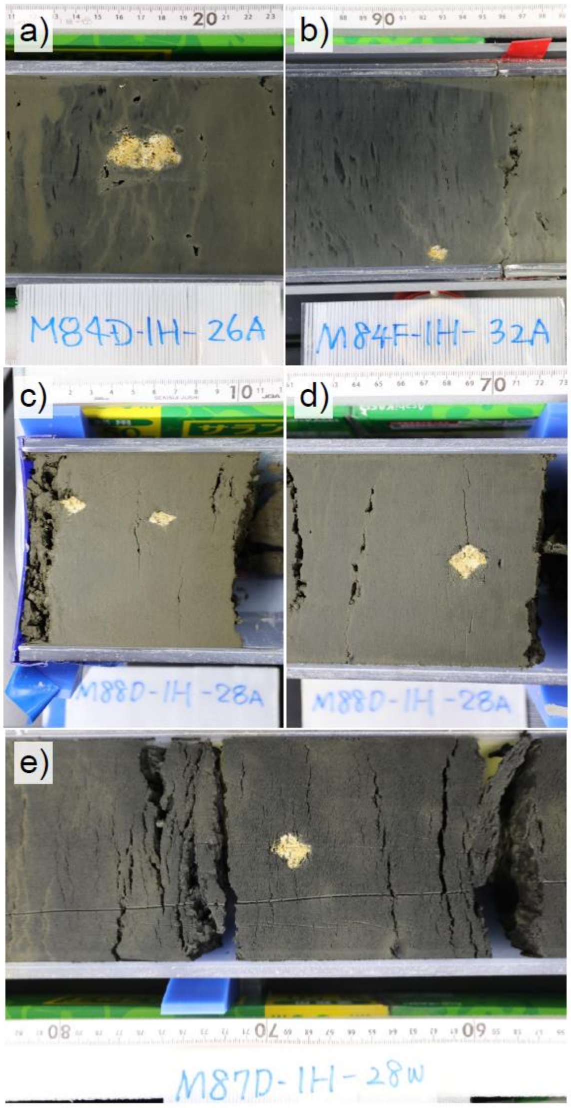

Supplementary Fig. 5. The observed authigenic carbonates (ikaites) in Sites M0084 (**a** & **b**), M0088 (**c** & **d**), and M0087 (**e**).

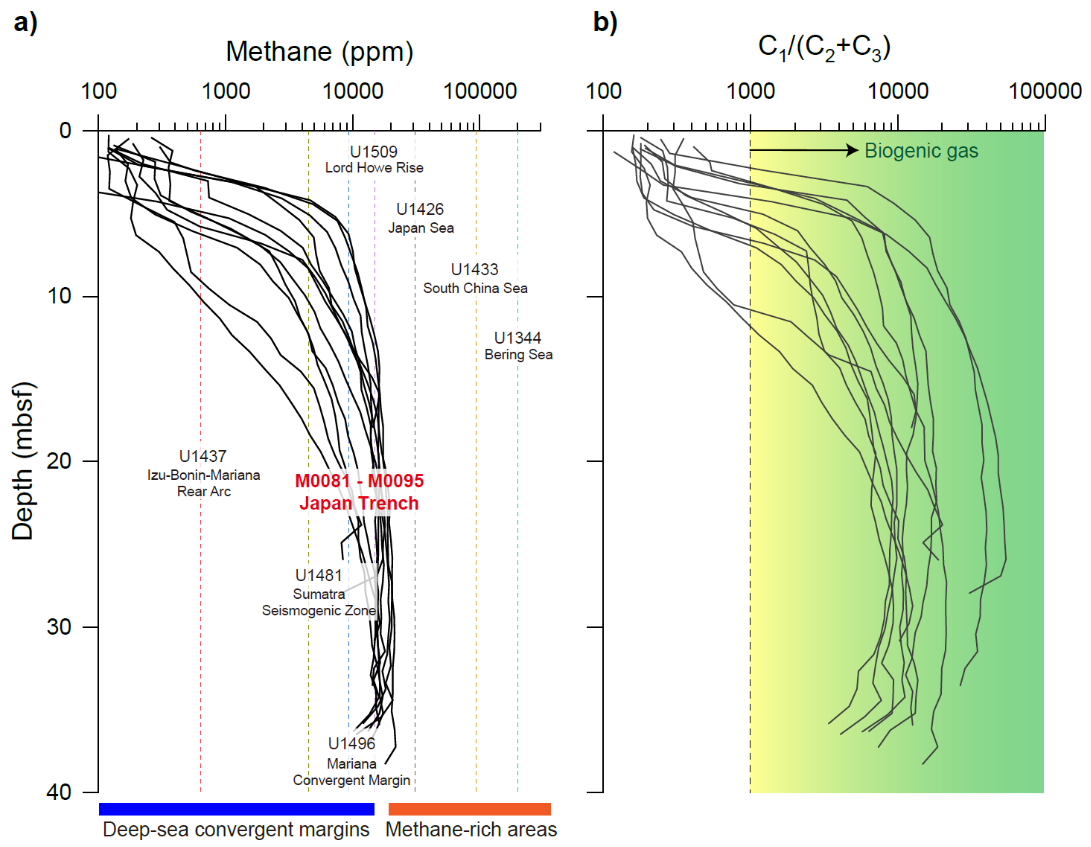

Supplementary Fig. 6. a, Headspace methane concentrations and **b,** Ratios of methane to ethane and propane (C_1 / (C_2 + C_3)) along the Japan Trench. Data are smoothed using adjacent-averaging method with 15 points of window. Dashed lines mark the maximum methane concentrations of the reference IODP Holes (from IODP database: https://iodp.tamu.edu/).

Supplementary Table 1. Summaries of IODP Holes used for geochemical analyses.

Site	Latitude (N)	Longitude (E)	Water depth (m)	Hole for analyses		
				IW geochemistry	Headspace	DOC and DIC
M0081	36°4.336′	142°44.14′	8020	D	F	D
M0082	36°6.050′	142°45.508′	7993	D	D	
M0083	38°45.413′	144°7.755′	7620	D	D	D
M0084	40°23.726′	144°25.328′	7590	D	F	D
M0086	39°46.756′	144°16.524′	7502	B	B	
M0088	40°5.586′	144°19.541′	7550	D	D	
M0089	38°43.202′	144°7.538′	7607	D	D	
M0090	38°17.834′	144°3.549′	7445	D	D	
M0091	37°24.747′	143°43.729′	7802	D	D	
M0092	36°54.672′	143°25.416′	7702	D	D	
M0093	39°4.909′	144°13.000′	7454	B	D	
M0095	36°53.501′	143°24.473′	7697	B	B	

Supplementary Table 2. Estimation of the converted DIC (expressed as fraction, *f* ranges from 0 to 1) in the deepest IW at sites M0081, M0083 and M0084.

Sites	IW depth (mbsf)	$f_{residual}$		$f_{converted}$	
		ε_c = –50‰	ε_c = –90‰	ε_c = –50‰	ε_c = –90‰
M0081	34.345	80%	88%	20%	12%
M0083	33.055	74%	85%	26%	15%
M0084	27.825	62%	76%	38%	24%

Supplementary Table 3. IW DOC concentrations of Holes M0081D, M0083D and M0084D.

Depth (mbsf)	DOC Concentration (mM)	Depth (mbsf)	DOC Concentration (mM)	Depth (mbsf)	DOC Concentration (mM)
**M0081D**		**M0083D**		**M0084D**	
0.175	2.285	0.270	1.692	0.350	1.721
1.175	2.884	1.560	1.871	1.995	1.876
2.185	4.106	2.570	2.011	3.365	1.531
3.190	4.097	3.580	2.220	5.515	1.863
6.705	4.158	5.095	2.337	7.575	3.030
11.735	7.998	7.115	2.334	9.595	5.751
15.850	12.138	9.135	2.498	11.670	6.889
17.865	11.631	10.145	2.462	13.690	7.767
19.985	8.418	12.155	2.907	15.715	9.859
22.085	9.284	14.185	4.350	17.745	7.174
24.170	12.125	16.230	4.640	19.755	8.188
26.245	11.427	18.310	5.644	21.775	7.260
28.290	11.997	20.330	7.436	23.790	7.443
30.310	7.298	22.480	5.545	25.790	7.033
32.320	9.039	24.515	5.696	27.825	6.847
34.345	8.197	26.565	4.610		
		28.590	4.587		
		30.975	5.476		
		33.055	6.121		
		35.075	6.683		

Supplementary Table 4. IW DIC concentrations of Holes M0081D, M0083D and M0084D.

Depth (mbsf)	DIC Concentration (mM)	Depth (mbsf)	DIC Concentration (mM)	Depth (mbsf)	DIC Concentration (mM)
**M0081D**		**M0083D**		**M0084D**	
0.175	42.20	0.270	18.52	1.370	22.45
2.185	30.11	1.560	27.66	3.365	29.60
4.695	39.42	2.570	30.07	6.565	44.75
8.720	43.60	3.580	33.84	11.670	46.15
15.850	42.50	5.095	34.95	13.690	52.34
19.985	44.42	7.115	26.92	19.755	36.23
26.245	44.56	9.135	30.54	23.790	35.94
30.310	49.97	20.330	37.41	27.825	61.22
34.345	46.19	24.515	43.02		
		28.590	46.14		
		33.055	49.29		

Supplementary Table 5. IW $\delta^{13}C_{DOC}$ values of Holes M0081D, M0083D and M0084D.

Depth (mbsf)	$\delta^{13}C_{DOC}$ (‰)	Depth (mbsf)	$\delta^{13}C_{DOC}$ (‰)	Depth (mbsf)	$\delta^{13}C_{DOC}$ (‰)
**M0081D**		**M0083D**		**M0084D**	
0.175	-18.62	0.270	-19.50	0.350	-19.07
1.175	-19.46	1.560	-19.51	1.995	-20.21
2.185	-18.70	2.570	-19.32	3.365	-18.76
3.190	-19.00	3.580	-19.16	5.515	-19.04
6.705	-18.51	5.095	-18.90	7.575	-18.35
11.735	-12.91	7.115	-19.04	9.595	-16.27
15.850	-12.23	9.135	-19.50	11.670	-13.31
17.865	-11.29	10.145	-15.11	13.690	-16.06
19.985	-13.87	12.155	-19.38	15.715	-21.86
22.085	-15.14	14.185	-17.85	17.745	-18.73
24.170	-12.46	16.230	-16.02	19.755	-16.66
26.245	-12.15	18.310	-15.87	21.775	-17.12
28.290	-14.36	20.330	-15.93	23.790	-18.94
30.310	-18.01	22.480	-17.15	25.790	-18.65
32.320	-15.76	24.515	-16.83	27.825	-18.95
34.345	-19.59	26.565	-18.77		
		28.590	-19.23		
		30.975	-19.33		
		33.055	-18.77		

Supplementary Table 6. IW $\delta^{13}C_{DIC}$ values of Holes M0081D, M0083D and M0084D.

Depth (mbsf)	$\delta^{13}C_{DIC}$ (‰)	Depth (mbsf)	$\delta^{13}C_{DIC}$ (‰)	Depth (mbsf)	$\delta^{13}C_{DIC}$ (‰)
**M0081D**		**M0083D**		**M0084D**	
0.175	-12.59	0.270	-14.13	1.370	-14.06
2.185	-14.43	1.560	-14.77	3.365	-14.76
4.695	-14.83	2.570	-15.06	6.565	-15.42
8.720	-14.22	3.580	-15.40	11.670	-1.050
15.850	-8.90	5.095	-15.56	13.690	3.380
19.985	-4.85	7.115	-13.52	19.755	8.880
26.245	-3.06	9.135	-14.45	23.790	10.15
30.310	-0.83	20.330	-5.91	27.825	10.60
34.345	-1.29	24.515	-2.80		
		28.590	-0.30		
		33.055	0.87		

Supplementary Table 7. IW $DO^{14}C$ and $DI^{14}C$ ages of Holes M0081D, M0083D and M0084D.

Depth (mbsf)	$DO^{14}C$ age (kyr)	$DI^{14}C$ age (kyr)	Depth (mbsf)	$DO^{14}C$ age (kyr)	$DI^{14}C$ age (kyr)	Depth (mbsf)	$DO^{14}C$ age (kyr)	$DI^{14}C$ age (kyr)
	**M0081D**			**M0083D**			**M0084D**	
0.175	2.10 ± 0.025	1.73 ± 0.02	0.270	2.72 ± 0.035	2.55 ± 0.02	0.350	1.97 ± 0.04	2.68 ± 0.03*
1.175	1.84 ± 0.02	1.88 ± 0.04*	1.560	3.35 ± 0.035	3.00 ± 0.02	1.370	2.61 ± 0.08*	2.94 ± 0.03
2.185	1.91 ± 0.02	2.04 ± 0.02	2.570	3.42 ± 0.045	3.10 ± 0.02	1.995	3.00 ± 0.04	3.10 ± 0.05*
3.190	2.12 ± 0.02	2.15 ± 0.04*	3.580	3.52 ± 0.040	3.35 ± 0.02	3.365	4.03 ± 0.035	3.45 ± 0.02
4.695	2.32 ± 0.04*	2.32 ± 0.02	5.095	3.56 ± 0.035	3.61 ± 0.02	5.515	4.41 ± 0.035	3.81 ± 0.04*
6.705	2.59 ± 0.02	2.57 ± 0.035*	7.115	3.66 ± 0.035	3.90 ± 0.025	6.565	4.31 ± 0.055*	3.98 ± 0.02
8.720	2.78 ± 0.035*	2.82 ± 0.015	9.135	4.64 ± 0.040	4.62 ± 0.025	7.575	4.21 ± 0.025	4.10 ± 0.04*
11.735	3.06 ± 0.015	3.18 ± 0.035*	10.145	4.81 ± 0.040	4.89 ± 0.055*	9.595	4.24 ± 0.03	4.34 ± 0.04*
15.850	3.31 ± 0.02	3.68 ± 0.02	12.155	5.08 ± 0.030	5.42 ± 0.055*	11.670	4.36 ± 0.02	4.59 ± 0.02
17.865	3.60 ± 0.02	4.01 ± 0.04*	14.185	5.01 ± 0.030	5.96 ± 0.055*	13.690	4.61 ± 0.025	4.92 ± 0.025
19.985	4.05 ± 0.02	4.36 ± 0.02	16.230	5.93 ± 0.030	6.51 ± 0.055*	15.715	5.04 ± 0.025	5.26 ± 0.05*
22.085	4.40 ± 0.02	4.79 ± 0.045*	18.310	5.91 ± 0.030	7.06 ± 0.055*	17.745	5.25 ± 0.025	5.60 ± 0.05*
24.170	4.74 ± 0.02	5.21 ± 0.045*	20.330	6.04 ± 0.030	7.60 ± 0.03	19.755	5.28 ± 0.025	5.94 ± 0.025
26.245	5.10 ± 0.02	5.63 ± 0.025	22.480	6.69 ± 0.030	8.19 ± 0.06*	21.775	5.93 ± 0.03	6.33 ± 0.05*
28.290	5.44 ± 0.02	6.02 ± 0.05*	24.515	7.54 ± 0.035	8.74 ± 0.03	23.790	6.12 ± 0.03	6.71 ± 0.025
30.310	6.17 ± 0.025	6.41 ± 0.025	26.565	8.81 ± 0.045	9.58 ± 0.07*	25.790	6.51 ± 0.035	7.07 ± 0.055*
32.320	6.48 ± 0.025	6.82 ± 0.055*	28.590	9.77 ± 0.05	10.40 ± 0.04	27.825	6.95 ± 0.035	7.43 ± 0.03
34.345	6.93 ± 0.03	7.24 ± 0.03	30.975	9.94 ± 0.05	11.25 ± 0.095*			
			33.055	10.30 ± 0.055	12.00 ± 0.055			
			35.075	11.30 ± 0.07	12.72 ± 0.055*			

*Note: Linearly extrapolated values used in Fig. 3 are marked by *.*

Supplementary Table 8. IW geochemical parameters of the studied IODP Sites.

Depth (mbsf)	Alkalinity (mM)	SO_4^{2-} (mM)	Depth (mbsf)	Alkalinity (mM)	SO_4^{2-} (mM)	Depth (mbsf)	Alkalinity (mM)	SO_4^{2-} (mM)
**M0081D**			**M0082D**			**M0083D**		
BW	2.53	28.80	0.40		21.09	BW	2.61	28.21
0.18	17.70	20.12	0.90	30.93	12.98	0.27	24.68	16.53
0.68	8.22	26.38	1.41	37.88	9.02	1.06	33.05	11.70
1.18	25.64	16.00	1.91	44.45	5.89	1.56	37.04	9.68
1.69	32.50	12.32	2.42	48.23	3.65	2.06	39.58	8.15
2.19	40.02	8.26	2.92	52.34	2.12	2.57	42.10	6.98
2.69	43.36	6.67	3.43	54.95	1.00	3.07	44.23	5.90
3.19	47.06	5.01	3.93	56.08	0.46	3.58	45.23	5.32
3.69	49.91	3.78	4.44	57.90	0.15	4.08	46.62	4.57
4.19	50.97	3.20	4.94	59.25	0.04	5.10	48.59	3.62
4.70	53.43	2.65	5.95	62.19	0.13	6.11	49.91	2.97
5.70	56.53	1.53	6.95	63.69	0.29	7.12	50.11	2.90
6.71	59.04	2.76	7.96	65.01	0.09	8.13	50.55	2.62
7.71	56.85	0.61	8.99	66.30	0.22	9.14	50.09	2.46
8.72	60.40	0.40	10.01	66.61	0.35	10.15	50.20	2.30
9.73	60.69	0.28	12.04	69.41	0.16	12.16	53.46	0.83
11.74	64.13	0.40	14.10	70.74	0.15	14.19	57.61	0.44
13.75	66.79	0.91	16.29	71.37	0.10	16.23	56.44	1.17
15.85	70.29	0.39	18.35	71.39	0.15	18.31	62.60	0.28
17.87	72.14	0.26	20.34	71.47	0.25	20.33	64.94	0.20
19.99	72.19	0.48	22.42	71.47	0.06	22.48	65.84	0.21
22.09	72.74	0.34	24.50	72.42	0.01	24.52	66.24	0.46

Continued

24.17	72.39	0.23	26.64	70.95	0.26	26.57	66.55	0.05
26.25	75.13	0.24	28.82	70.73	0.20	28.59	67.31	0.14
28.29	73.28	0.26	30.84	70.26	0.11	30.98	68.01	0.02
30.31	73.61	0.37	33.07	71.26	0.02	33.06	69.49	0.01
32.32	73.43	0.21	35.12	70.99		35.08	71.92	0.04
34.35	72.03	0.32						

**Depth (mbsf)**	**Alkalinity (mM)**	**SO_4^{2-} (mM)**	**Depth (mbsf)**	**Alkalinity (mM)**	**SO_4^{2-} (mM)**	**Depth (mbsf)**	**Alkalinity (mM)**	**SO_4^{2-} (mM)**
**M0084D**			**M0086B**			**M0088D**		
BW	2.70	28.39	0.11	16.68	21.26	0.45	25.89	16.61
0.35	13.42	23.04	0.61	17.06	21.37	0.95	33.65	12.53
0.85	23.50	17.67	1.10	30.01	14.27	1.52	37.88	10.29
1.37	30.37	14.92	1.60	31.67	12.70	2.02	41.16	8.47
2.00	35.70	12.21	2.12	32.58	12.05	2.52	44.81	6.47
2.36	37.37	11.55	2.62	33.94	10.51	3.02	48.12	4.49
2.74	38.97	10.72	3.12	35.91	10.30	3.53	51.12	3.57
3.37	40.94	9.74	3.62	37.76	9.03	4.03	54.62	1.36
4.38	46.77	6.30	4.13	39.34	8.12	4.54	57.54	0.35
5.52	53.45	3.06	4.63	41.20	7.32	5.04	59.30	0.09
6.57	60.15	0.32	5.63	44.78	5.69	6.06	61.90	0.16
7.58	62.68	0.25	6.64	12.20	23.23	7.15	64.39	0.15
8.59	65.84	0.10	7.64	50.99	2.99	8.15	67.13	0.13
9.60	69.20	0.34	8.65	54.87	1.50	9.16	68.61	0.12
11.67	74.95	0.14	9.66	57.55	0.56	10.17	69.67	0.15
13.69	81.23	0.13	10.67	59.37	0.31	12.18	73.14	0.46
15.72	85.97	0.15	12.70	62.45	0.24	14.19	79.27	0.26

Continued

17.75	92.19	0.05	14.75	64.76	0.09	16.20	82.86	0.17
19.76	94.12	0.28	16.79	67.16	0.07	18.26	88.36	0.16
21.78	96.45	0.24				20.23	92.59	0.10
23.79	97.86	0.12				22.27	92.90	0.15
25.79	94.50	0.08				24.32	97.07	0.12
27.83	95.38					26.37	96.14	0.06
29.83	88.61	0.01				28.38	95.16	0.12
31.90	84.74					30.21	92.50	
33.91	79.49	0.01				32.76	88.83	
						34.77	85.90	

**Depth (mbsf)**	**Alkalinity (mM)**	**SO_4^{2-} (mM)**	**Depth (mbsf)**	**Alkalinity (mM)**	**SO_4^{2-} (mM)**	**Depth (mbsf)**	**Alkalinity (mM)**	**SO_4^{2-} (mM)**
**M0089D**			**M0090D**			**M0091D**		
0.35	11.51	23.39	0.35	21.48	16.93	0.10	7.63	25.84
0.85	20.56	17.49	0.71	21.30	16.80	0.60	11.94	22.51
1.36	24.77	14.39	1.21	30.31	10.84	1.10	18.70	19.80
1.86	29.61	11.84	1.71	34.99	7.77	1.62	22.18	17.14
2.37	31.45	10.32	2.21	39.37	4.95	2.12	26.13	15.45
2.87	33.41	9.00	2.72	41.06	3.98	2.63	27.29	14.82
3.38	36.06	7.76	3.22	44.23	2.37	3.13	29.56	13.47
3.88	37.54	6.35	3.73	47.18	1.06	3.63	31.55	12.44
4.39	39.23	5.22	4.23	47.99	0.56	4.13	34.26	10.99
4.89	41.21	3.61	5.23	51.48	0.15	5.14	38.22	9.53
5.90	46.74	1.57	6.24	52.82	0.15	6.15	40.85	8.38
6.90	48.90	0.56	7.30	53.57	0.48	7.15	41.96	7.75
7.91	50.73	0.15	8.32	55.08	0.35	8.16	42.75	7.10

Continued

8.92	51.84	0.16	9.36	50.83	2.63	9.17	45.10	5.91
9.93	53.61	0.22	11.38	58.86	0.35	11.19	51.52	2.74
11.96	55.12	0.21	13.44	61.21	0.20	13.21	60.48	0.31
14.01	57.77	0.11	15.51	63.61	0.09	15.24	63.20	0.41
16.12	59.10	0.09	17.54	64.86	0.08	17.25	64.66	0.30
18.22	61.06	0.14	19.77	65.82	0.24	19.28	67.31	0.06
20.33	62.07	0.05	21.86	67.62	0.16	21.30	68.29	0.12
22.48	64.26	0.20	23.97	69.55	0.15	23.32	68.14	0.25
24.60	65.29	0.23	26.10	67.85	0.01	25.31	68.86	0.25
26.80	66.07	0.26	28.22	70.12		27.50	69.33	0.23
28.92	66.28	0.01	30.30	70.37		29.65	67.72	0.67
31.10	67.78	0.01	32.39	70.81				
33.26	68.50							
35.31	69.27							

**Depth (mbsf)**	**Alkalinity (mM)**	**SO_4^{2-} (mM)**	**Depth (mbsf)**	**Alkalinity (mM)**	**SO_4^{2-} (mM)**	**Depth (mbsf)**	**Alkalinity (mM)**	**SO_4^{2-} (mM)**
	**M0092D**			**M0093B**			**M0095B**	
0.21	7.07	25.42	0.33	18.19	20.22	0.61	17.69	18.46
0.71		20.48	0.83	22.73	18.26	1.11	24.66	14.87
1.23	23.88	15.40	1.33	24.98	17.13	1.62	28.99	12.48
1.73	27.82	13.58	1.83	29.33	14.97	2.12	32.37	10.43
2.23	31.71	11.20	2.33	31.30	13.84	2.62	36.71	8.28
2.73	34.56	9.36	2.84	32.69	13.39	3.12	39.37	6.46
3.24	37.21	7.82	3.34	35.06	11.97	3.63	42.28	5.14
3.74	39.02	6.70	3.85	36.74	11.30	4.13	44.41	4.04
4.25	40.86	5.82	4.35	37.40	11.40	5.13	47.28	3.11

Continued

4.76	41.88	4.95	5.35	38.04	11.13	6.14	53.84	0.26
5.76	44.00	3.56	6.36	37.75	11.08	7.14	56.89	0.20
6.77	47.41	2.12	7.37	36.96	11.06	8.14	58.22	0.03
7.78	49.52	0.65	8.38	37.73	10.39	9.16	60.11	0.06
8.78	52.30	0.12	9.38	37.92	9.68	10.16	60.76	0.13
9.79	54.68	0.07	10.39	38.98	8.44	12.19	63.10	0.04
11.79	55.79	0.09	12.39	45.19	4.54	14.20	64.44	0.17
13.82	57.33	0.69	14.41	53.55	1.13	16.30	65.53	0.25
15.84	57.62	0.91	16.42	53.41	2.52	18.39	65.84	0.22
17.89	60.69	0.12	18.46	61.73	0.24	20.50	67.13	0.03
19.96	60.41	0.14	20.51	63.77	0.12	22.63	68.74	
22.05	59.10	0.51	22.61	65.88	0.01	24.75	69.07	0.01
24.15	60.49	0.01	24.76	66.97	0.08	26.76	69.06	0.04
26.25	59.79	0.01						
28.33	55.55							
30.45	57.40							
32.54	56.57							

Supplementary Table 9. Headspace gas concentrations of the studied IODP Sites.

Depth (mbsf)	Methane (ppm)	Ethane (ppm)	Propane (ppm)	Methane (mM)	$C_1/(C_2+C_3)$
		**M0081F**			
1.07	135.61	0.49	0.37	0.03	158
2.02	161.87	0.62	0.48	0.04	147
3.03	216.16	0.61	0.51	0.05	193
4.05	150.86	0.44	0.34	0.04	195
5.06	149.42	0.53	0.44	0.04	154
6.06	225.02	0.52	0.43	0.06	238
7.08	227.26	0.54	0.44	0.06	232
8.09	476.09	0.47	0.35	0.12	577
9.09	778.93	0.54	0.42	0.19	809
10.10	1268.00	0.70	0.59	0.31	983
11.11	2272.10	0.54	0.43	0.56	2345
12.12	3825.65	0.60	0.50	0.95	3493
13.13	6123.59	0.70	0.59	1.51	4732
14.13	8246.61	0.73	0.63	2.00	6061
15.15	4010.07	0.56	0.45	0.93	3971
16.20	7343.16	0.62	0.52	1.74	6449
17.25	11736.92	0.65	0.54	2.87	9818
18.29	8461.78	0.62	0.49	2.05	7636
19.34	10775.88	0.56	0.46	2.62	10633
20.40	8044.30	0.51	0.42	1.92	8671
21.45	5887.26	0.56	0.46	1.42	5760
22.50	10733.84	0.65	0.57	2.53	8853

Continued

23.59	12808.39	0.53	0.45	3.08	13025
24.68	7771.12	0.85	0.81	2.02	4658
25.77	6963.16	0.83	0.75	1.84	4392
26.83	6292.99	0.70	0.58	1.36	4909
27.89	16804.91	0.64	0.54	4.04	14312
28.94	10471.45	0.56	0.46	2.56	10218
30.04	22096.76	0.61	0.51	5.44	19577
31.12	21046.27	0.48	0.42	5.18	23466
32.17	6398.92	0.83	0.72	1.56	4128
33.22	17547.78	0.40	0.30	4.45	25291
34.23	23556.55	0.92	0.79	5.97	13718
35.24	12541.68	0.81	0.69	3.30	8357
36.25	20261.72	0.95	0.91	5.39	10873
37.29	13158.67	0.93	0.85	3.32	7402
**Depth (mbsf)**	**Methane (ppm)**	**Ethane (ppm)**	**Propane (ppm)**	**Methane (mM)**	**$C_1/(C_2+C_3)$**
		**M0082D**			
1.06	119.10	0.34	0.28	0.03	193
2.07	188.30	0.36	0.30	0.05	285
3.08	317.57	0.41	0.31	0.08	442
4.09	1328.18	0.50	0.40	0.32	1473
5.10	3543.37	0.85	0.78	0.89	2175
6.10	5728.28	0.60	0.50	1.34	5216
7.11	8147.69	0.47	0.40	1.89	9400
8.12	8409.59	0.55	0.46	2.00	8359
9.14	13560.98	0.49	0.43	3.22	14702

Continued

10.17	10317.81	0.44	0.37	2.40	12760
11.19	17650.17	0.31	0.25	4.46	31494
12.21	14418.14	0.65	0.58	3.53	11808
13.22	11600.49	0.61	0.52	2.78	10252
14.26	14703.98	0.44	0.32	3.46	19220
15.30	12098.20	0.65	0.55	2.76	10041
16.34	16831.11	0.48	0.39	4.14	19339
17.36	14363.99	0.64	0.55	3.40	12020
18.40	20713.67	0.73	0.64	4.71	15172
19.44	5330.24	0.64	0.54	1.25	4496
20.49	12990.37	1.07	0.92	3.00	6515
21.55	17219.19	0.75	0.64	4.02	12342
22.57	18374.30	0.59	0.51	4.33	16709
23.60	9485.97	0.84	0.77	2.32	5896
24.66	17699.55	0.81	0.70	4.23	11727
25.71	4769.92	0.81	0.68	1.19	3210
26.80	20263.13	0.80	0.70	5.05	13455
27.91	24402.73	1.10	0.99	5.84	11660
28.97	21699.67	0.91	0.76	5.18	12987
30.04	16081.24	0.56	0.47	3.93	15654
31.12	5621.42	0.92	0.77	1.36	3327
32.17	20247.50	0.66	0.58	4.90	16319
33.23	19858.59	0.78	0.66	4.97	13831
34.24	16370.77	0.84	0.69	3.90	10680
35.29	14348.44	0.86	0.68	3.50	9259

Continued

36.35	10149.19	0.98	0.79	2.49	5741
**Depth (mbsf)**	**Methane (ppm)**	**Ethane (ppm)**	**Propane (ppm)**	**Methane (mM)**	**$C_1/(C_2+C_3)$**
			**M0083D**		
0.41	260.22	0.80	0.65	0.06	179
1.21	515.91	0.99	0.78	0.13	292
2.22	376.51	0.73	0.58	0.10	288
3.23	263.78	0.48	0.36	0.07	311
4.25	369.34	0.71	0.54	0.09	295
5.26	411.78	0.84	0.64	0.10	278
6.27	374.67	0.73	0.57	0.10	288
7.28	286.32	0.75	0.59	0.07	214
8.29	399.19	0.81	0.63	0.10	278
9.30	1647.49	0.43	0.33	0.39	2165
10.30	2874.64	0.45	0.32	0.72	3703
11.31	4465.27	0.53	0.38	1.12	4882
12.31	4708.52	0.46	0.34	1.18	5872
13.33	6240.15	0.66	0.48	1.59	5484
14.34	9989.43	0.53	0.35	2.34	11448
15.37	5943.35	2.00	2.09	1.41	1453
16.44	5963.29	2.34	2.47	1.53	1239
17.45	8857.46	2.25	2.41	2.41	1901
18.47	5689.59	1.00	0.98	1.37	2875
19.48	9900.06	2.74	3.05	2.64	1708
20.56	5183.22	0.64	0.56	1.21	4335
21.63	19692.16	0.84	0.71	4.55	12703

Continued

22.66	13225.49	1.01	0.91	3.15	6867
23.67	11043.92	0.57	0.45	2.53	10863
24.68	17388.11	0.67	0.53	4.17	14513
25.72	10244.94	0.62	0.51	2.49	9072
26.74	18823.22	0.66	0.52	4.77	16052
27.74	18035.89	3.07	3.58	4.71	2710
28.75	12953.27	3.27	3.89	3.68	1810
29.76	17284.90	0.89	0.71	4.12	10792
31.12	10548.72	0.69	0.57	2.72	8419
32.21	18177.04	1.30	1.20	4.57	7272
33.21	19256.86	0.92	0.73	4.78	11689
34.23	21933.69	1.10	0.90	5.43	10960
35.23	13750.42	0.75	0.62	3.47	10102
36.32	13207.47	1.13	0.96	3.30	6338
**Depth (mbsf)**	**Methane (ppm)**	**Ethane (ppm)**	**Propane (ppm)**	**Methane (mM)**	**$C_1/(C_2+C_3)$**
		**M0084F**			
0.95	304.04	0.43	0.31	0.07	412
1.89	544.78	0.39	0.26	0.13	843
2.42	256.35	0.46	0.31	0.05	334
3.43	783.83	0.83	0.65	0.20	529
4.44	990.61	0.91	0.71	0.26	613
5.45	1963.63	0.97	0.74	0.51	1146
6.47	9186.69	0.90	0.69	2.29	5793
7.51	15351.80	0.92	0.68	3.68	9628
8.57	14747.94	0.98	0.72	3.52	8649

Continued

9.63	20474.15	0.91	0.69	4.93	12818
10.72	18852.46	0.85	0.62	4.47	12835
11.81	12258.48	0.99	0.77	2.97	6959
12.84	18241.50	0.78	0.59	4.40	13371
13.85	14421.42	0.94	0.72	3.51	8707
14.86	18717.72	1.00	0.83	4.72	10236
15.87	17324.15	0.97	0.76	4.26	10047
16.88	14953.70	0.89	0.74	4.26	9158
17.90	13150.53	0.93	0.76	3.40	7774
18.90	6924.64	1.66	1.39	1.92	2270
19.95	19696.86	0.40	0.33	4.31	27134
21.00	14540.00	0.47	0.40	3.45	16752
22.04	16415.30	0.65	0.53	3.77	13873
23.06	24660.54	0.58	0.50	5.37	22919
24.11	20711.54	0.47	0.42	4.66	23292
25.17	29633.45	0.40	0.32	6.71	40715
26.25	18779.60	0.72	0.59	4.05	14298
27.31	12464.10	0.81	0.68	2.96	8378
28.37	28997.19	0.47	0.40	6.54	33374
29.37	18898.15	0.39	0.32	4.31	26832
30.37	24938.23	0.52	0.41	6.07	26994
31.43	18933.95	0.60	0.47	4.43	17821
32.50	16576.06	0.46	0.35	3.97	20462
34.06	15148.18	0.66	0.57	3.78	12310
35.12	25917.57	0.86	0.72	6.16	16359

Continued

36.18	22840.60	0.52	0.44	5.64	23828
37.26	24873.85	0.78	0.66	6.16	17347
38.31	17948.24	0.68	0.54	4.51	14705
**Depth (mbsf)**	**Methane (ppm)**	**Ethane (ppm)**	**Propane (ppm)**	**Methane (mM)**	**$C_1/(C_2+C_3)$**
			**M0086B**		
0.75	186.02	0.57	0.47	0.04	180
1.77	253.35	0.75	0.61	0.06	187
2.77	237.97	0.77	0.61	0.06	172
3.78	176.39	0.68	0.51	0.04	148
4.78	196.43	0.54	0.42	0.05	204
5.79	761.92	1.84	1.90	0.19	204
6.79	160.09	0.50	0.33	0.04	192
7.80	539.45	0.85	0.68	0.14	353
8.81	607.81	0.96	0.80	0.16	347
9.82	956.23	0.79	0.60	0.25	686
10.83	2794.71	0.89	0.68	0.71	1788
11.84	7007.41	0.99	0.77	1.77	3987
12.86	10731.35	0.79	0.63	2.70	7541
13.90	16965.09	0.70	0.51	4.31	13951
14.93	18677.69	0.97	0.75	4.98	10857
15.94	15181.19	0.51	0.39	4.12	16867
16.94	16653.42	0.87	0.70	4.82	10559
17.96	14476.48	0.67	0.50	4.33	12419

Continued

Depth (mbsf)	Methane (ppm)	Ethane (ppm)	Propane (ppm)	Methane (mM)	$C_1/(C_2+C_3)$
		**M0088D**			
1.10	138.86	0.46	0.32	0.03	178
2.17	564.91	0.75	0.63	0.14	408
3.18	668.45	1.03	0.80	0.17	366
4.19	937.34	0.87	0.69	0.24	601
5.20	4555.71	0.97	0.74	1.18	2656
6.22	11504.37	1.07	0.84	2.82	6038
7.30	14106.44	0.38	0.28	3.47	21440
8.31	15518.82	0.66	0.51	3.85	13202
9.32	17517.88	1.15	0.90	4.22	8578
10.32	14495.17	0.51	0.42	3.48	15579
11.33	22379.94	0.61	0.51	5.31	19856
12.33	14351.33	0.88	0.74	3.41	8824
13.34	14323.07	0.76	0.63	3.36	10302
14.34	12689.75	1.77	1.48	3.48	3900
15.35	12728.81	0.24	0.18	2.85	30159
16.36	14302.03	0.57	0.50	3.17	13416
17.41	16379.41	0.53	0.47	3.72	16485
18.37	19154.57	0.46	0.38	4.32	22882
19.38	18628.37	0.58	0.53	4.09	16806
20.38	20176.38	0.43	0.36	4.37	25457
21.39	16824.10	0.94	0.78	3.81	9820
22.39	13888.02	0.30	0.24	3.17	25853
23.40	13851.89	0.36	0.26	3.15	22372

Continued

24.47	16752.67	0.46	0.37	3.81	20063
25.52	14154.46	0.53	0.43	3.26	14784
26.52	20269.85	0.64	0.53	4.70	17311
27.53	19588.51	0.45	0.34	4.78	24892
28.54	14898.15	0.99	0.83	3.36	8160
29.40	9250.76	0.80	0.67	2.10	6308
30.36	11461.77	0.65	0.51	2.59	9846
31.91	14528.43	0.64	0.49	3.69	12831
32.91	16053.13	0.63	0.55	3.94	13692
33.92	15191.41	0.62	0.50	3.74	13552
34.92	14616.20	0.76	0.63	3.70	10492
35.95	16230.85	0.71	0.57	3.83	12653
**Depth (mbsf)**	**Methane (ppm)**	**Ethane (ppm)**	**Propane (ppm)**	**Methane (mM)**	**$C_1/(C_2+C_3)$**
			**M0089D**		
1.01	119.32	0.44	0.31	0.03	158
2.02	440.27	0.67	0.55	0.11	360
3.03	107.58	0.52	0.40	0.03	117
4.04	203.33	0.58	0.43	0.05	202
5.05	2766.88	3.42	3.77	0.80	384
6.05	331.50	0.51	0.42	0.08	355
7.06	1215.15	0.60	0.40	0.29	1221
8.07	3241.60	0.65	0.49	0.77	2846
9.08	5278.70	0.58	0.42	1.24	5273
10.09	6759.82	0.61	0.46	1.60	6339
11.11	9489.57	0.89	0.76	2.31	5742

Continued

12.12	10268.93	1.67	1.77	2.63	2985
13.14	12341.34	0.93	0.81	3.02	7100
14.18	7368.08	0.57	0.47	1.93	7059
15.23	10536.53	0.79	0.62	2.55	7476
16.28	16855.80	0.99	0.81	4.18	9365
17.33	18290.76	1.53	1.53	4.37	5988
18.38	21555.74	0.96	0.74	5.18	12709
19.43	21168.18	0.96	0.73	5.13	12466
20.50	12035.09	0.61	0.44	2.94	11374
21.58	15697.20	1.06	0.82	3.79	8340
22.65	25303.05	1.03	0.82	6.12	13682
23.71	24713.77	0.99	0.85	6.16	13390
24.76	24574.47	1.12	0.96	6.17	11842
25.85	13752.34	0.98	0.77	3.46	7852
26.95	19523.88	0.99	0.94	5.88	10123
28.02	29085.64	1.74	1.58	7.35	8758
29.07	23857.76	1.77	1.53	5.82	7239
30.11	18618.81	1.36	1.20	4.74	7269
31.25	5523.76	1.89	1.68	1.47	1550
32.36	27589.09	1.25	1.18	6.95	11325
33.41	25361.14	1.44	1.22	6.75	9524
34.45	20177.16	1.77	1.49	5.16	6194
35.46	19716.64	1.42	1.20	5.06	7519
36.50	10678.95	1.41	1.21	2.77	4083

Continued

Depth (mbsf)	Methane (ppm)	Ethane (ppm)	Propane (ppm)	Methane (mM)	$C_1/(C_2+C_3)$
			**M0090D**		
0.51	53.21	0.14	0.07	0.01	246
1.36	90.81	0.20	0.11	0.03	290
2.37	63.34	0.14	0.06	0.02	310
3.38	306.13	0.17	0.07	0.07	1315
4.38	1175.10	0.20	0.11	0.28	3787
5.39	3656.40	0.17	0.09	0.87	14023
6.44	6731.70	0.19	0.10	1.58	23451
7.45	839.87	0.08	0.05	0.20	6328
8.47	10254.30	0.20	0.10	2.39	34274
9.51	9514.64	0.19	0.12	2.31	30630
10.52	9234.06	0.18	0.13	2.24	29785
11.53	11290.26	0.22	0.14	2.61	31387
12.54	11173.40	0.18	0.11	2.57	38372
13.59	6399.23	0.16	0.11	1.57	23601
14.60	6792.13	0.18	0.13	1.67	21861
15.66	5017.25	0.15	0.10	1.37	20057
16.72	15205.46	0.21	0.13	3.60	43760
17.77	5070.40	0.19	0.11	1.26	16825
18.84	22851.42	0.26	0.17	5.60	53163
19.92	16903.20	0.22	0.14	4.03	46681
20.97	20770.65	0.27	0.20	5.07	44229
22.01	21923.07	0.24	0.15	5.28	55957
23.06	3618.06	0.16	0.11	0.90	13495

Continued

24.12	27079.45	0.28	0.17	6.72	60808
25.19	24376.38	0.24	0.16	6.20	60368
26.24	11115.55	0.22	0.17	2.80	28666
27.28	21330.15	0.26	0.18	5.20	48504
28.34	4820.56	0.18	0.15	1.20	14938
29.39	22402.14	0.28	0.19	5.62	48317
30.44	23396.06	0.33	0.26	5.83	39909
31.48	11923.23	0.26	0.19	2.98	26638
32.52	18031.25	0.30	0.25	4.53	32551
33.56	14295.70	0.30	0.23	3.77	26579

**Depth (mbsf)**	**Methane (ppm)**	**Ethane (ppm)**	**Propane (ppm)**	**Methane (mM)**	**$C_1/(C_2+C_3)$**
			**M0091D**		
0.25	120.82	0.42	0.32	0.03	162
1.27	152.71	0.52	0.42	0.04	162
2.28	86.86	0.35	0.26	0.02	143
3.28	276.12	0.74	0.60	0.07	205
4.29	237.00	0.49	0.37	0.06	275
5.30	385.08	0.73	0.57	0.10	295
6.30	151.61	0.44	0.27	0.04	214
7.31	153.04	0.55	0.45	0.04	153
8.32	129.14	0.45	0.35	0.03	162
9.33	119.37	0.38	0.27	0.03	184
10.34	165.12	0.39	0.29	0.04	243
11.34	201.52	0.39	0.28	0.05	299
12.35	392.05	0.44	0.34	0.10	501

Continued

13.36	632.08	0.38	0.28	0.16	955
14.38	1279.94	0.60	0.53	0.32	1134
15.39	1415.96	0.72	0.63	0.35	1049
16.40	2141.26	0.64	0.57	0.53	1777
17.41	2371.86	0.46	0.37	0.59	2849
18.42	3771.78	0.65	0.56	0.94	3121
19.43	3531.08	0.47	0.37	0.87	4222
20.44	6712.57	0.56	0.44	1.71	6722
21.45	7351.50	0.71	0.60	1.87	5648
22.46	9751.88	0.52	0.41	2.40	10489
23.48	9116.11	0.78	0.67	2.28	6306
24.51	10900.34	0.71	0.57	2.73	8545
25.55	9889.71	0.47	0.36	2.43	11955
26.61	15149.57	0.49	0.38	3.71	17445
27.65	13656.73	0.76	0.59	3.36	10055
28.74	17242.11	0.79	0.72	4.37	11425
29.80	17219.38	0.68	0.55	4.43	14081
30.86	14798.68	0.75	0.69	3.64	10255
**Depth (mbsf)**	**Methane (ppm)**	**Ethane (ppm)**	**Propane (ppm)**	**Methane (mM)**	**$C_1/(C_2+C_3)$**
		**M0092D**			
0.87	131.64	0.39	0.30	0.03	191
1.88	308.92	0.68	0.56	0.07	249
2.89	397.34	0.71	0.56	0.10	313
3.90	243.44	0.54	0.44	0.06	249
4.91	296.01	0.52	0.43	0.07	311

Continued

5.91	309.55	0.38	0.28	0.08	471
6.92	472.79	0.35	0.27	0.12	753
7.93	808.50	0.44	0.35	0.20	1020
8.94	1872.80	0.47	0.36	0.44	2254
9.94	2703.50	0.74	0.66	0.65	1921
10.94	4459.51	0.59	0.44	1.05	4355
11.95	5359.40	0.81	0.66	1.26	3624
12.96	6733.69	0.95	0.83	1.59	3770
13.97	8709.27	0.86	0.69	2.06	5628
14.97	7564.01	0.75	0.60	1.73	5609
15.99	10202.69	0.79	0.62	2.35	7248
17.02	13113.89	0.85	0.65	3.09	8741
18.04	12708.22	1.10	0.89	2.96	6393
19.09	8720.27	0.86	0.70	2.15	5583
20.12	15882.06	0.78	0.61	4.14	11456
21.17	16769.63	0.97	0.81	3.97	9429
22.21	15164.04	0.97	0.81	3.69	8493
23.27	19992.78	1.22	0.96	4.75	9199
24.32	24915.15	1.13	0.88	5.85	12359
25.35	18030.44	1.15	0.92	4.32	8720
26.41	24312.96	1.06	0.78	6.04	13211
27.45	26109.81	1.24	1.03	6.73	11492
28.48	21714.26	0.99	0.73	5.38	12660
29.54	20410.43	1.31	1.03	5.05	8702
30.60	8679.96	1.76	1.47	2.23	2683

Continued

31.66	24101.35	2.02	1.74	6.30	6424
32.69	19356.80	1.74	1.56	5.00	5857
33.75	20236.38	2.05	1.69	5.12	5411
34.81	15381.45	1.58	1.33	3.78	5283
35.86	12082.66	1.89	1.68	3.17	3384

**Depth (mbsf)**	**Methane (ppm)**	**Ethane (ppm)**	**Propane (ppm)**	**Methane (mM)**	**$C_1/(C_2+C_3)$**
			**M0093B**		
0.48	174.53	0.28	0.21	0.04	352
1.48	61.53	0.19	0.11	0.01	206
2.49	119.30	0.19	0.14	0.03	365
3.50	126.97	0.24	0.18	0.03	304
4.50	131.68	0.25	0.18	0.04	307
5.51	158.80	0.28	0.20	0.05	331
6.52	79.73	0.22	0.15	0.02	214
8.53	512.08	0.53	0.45	0.14	524
9.54	576.90	0.56	0.51	0.16	535
10.54	1183.96	0.90	0.86	0.35	674
11.54	1232.15	0.93	0.93	0.40	662
12.55	1019.92	0.86	0.80	0.31	617
13.56	675.71	0.84	0.91	0.20	386
14.57	755.84	0.59	0.54	0.21	674
15.57	1242.15	0.62	0.58	0.39	1032
16.58	3275.59	0.79	0.79	0.98	2073
17.59	4983.86	0.84	0.92	1.47	2823
18.61	10570.46	0.34	0.28	2.63	17109

Continued

19.63	7884.28	0.43	0.37	2.14	9890
20.67	6649.73	0.28	0.21	1.67	13551
21.73	18080.59	0.23	0.15	4.34	47748
22.76	15799.70	0.84	1.00	4.20	8575
23.83	8078.01	0.29	0.23	2.03	15440
24.90	8140.27	0.41	0.39	2.14	10148
25.96	8380.37	0.25	0.19	2.14	18871
**Depth (mbsf)**	**Methane (ppm)**	**Ethane (ppm)**	**Propane (ppm)**	**Methane (mM)**	**$C_1/(C_2+C_3)$**
			**M0095B**		
1.27	18.00	0.10	0.05	0.00	118
2.27	30.05	0.09	0.07	0.01	183
3.28	42.92	0.11	0.08	0.01	225
4.28	41.86	0.09	0.07	0.01	265
5.29	83.54	0.10	0.08	0.02	485
6.29	425.74	0.11	0.09	0.11	2113
7.29	1390.83	0.12	0.10	0.34	6308
8.31	2803.71	0.12	0.09	0.66	13162
9.31	4756.80	0.14	0.09	1.14	20163
10.32	5596.35	0.15	0.09	1.31	23192
11.33	7391.13	0.17	0.11	1.74	26310
12.34	8054.22	0.19	0.09	1.89	27953
13.34	10508.71	0.18	0.12	2.51	34420
14.37	12850.46	0.19	0.12	2.99	42014
15.40	12887.53	0.20	0.12	3.07	39662
16.45	11286.13	0.19	0.13	2.65	35289

Continued

17.50	13278.03	0.19	0.11	3.21	44181
18.54	19355.96	0.22	0.14	4.53	53554
19.61	21836.10	0.26	0.16	5.13	52140
20.66	17704.21	0.23	0.14	4.19	48052
21.71	22266.47	0.25	0.14	5.16	57209
22.78	13630.07	0.20	0.10	3.24	45467
23.86	21312.78	0.23	0.12	5.01	61466
24.90	18440.35	0.20	0.11	4.37	59028
25.91	19054.28	0.18	0.11	4.75	64270
26.92	19212.35	0.21	0.14	4.65	55164
27.95	8191.22	0.16	0.11	1.99	30693

Supplementary Table 10. BW DOC data of Holes M0081C, M0083C and M0084C used in Supplementary Fig. 1.

Hole	$\delta^{13}C_{DOC}$ (‰)	$\Delta^{14}C_{DOC}$ (‰)
M0081C	-29.82	-516.22
M0083C	-25.90	-504.23
M0084C	-26.03	-508.07

Supplementary references

1. Shan, S. *et al.* Carbon cycling in the deep Mariana Trench in the western north Pacific Ocean: Insights from radiocarbon proxy data. *Deep. Res. Part I Oceanogr. Res. Pap.* **164**, 103370 (2020).
2. Ishiwatari, R. *et al.* Source of Organic Matter in Sinking Particles in the Japan Trench: Molecular Composition and Carbon Isotopic Analyses. in *Dynamics and Characterization of Marine Organic Matter* 141–168 (2000).
3. Schwestermann, T. *et al.* Event-dominated transport, provenance, and burial of organic carbon in the Japan Trench. *Earth Planet. Sci. Lett.* **563**, 116870 (2021).
4. Bao, R. *et al.* Tectonically-triggered sediment and carbon export to the Hadal zone. *Nat. Commun.* **9**, 121 (2018).
5. McCorkle, D. C. & McNichol, A. P. The Stable Carbon Isotopic Composition of the Oceans. in *Encyclopedia of Ocean Sciences* 329–332 (Elsevier, 2019).
6. Wegener, G., Niemann, H., Elvert, M., Hinrichs, K. U. & Boetius, A. Assimilation of methane and inorganic carbon by microbial communities mediating the anaerobic oxidation of methane. *Environ. Microbiol.* **10**, 2287–2298 (2008).
7. Bradbury, H. J. & Turchyn, A. V. Reevaluating the carbon sink due to sedimentary carbonate formation in modern marine sediments. *Earth Planet. Sci. Lett.* **519**, 40–49 (2019).
8. Pohlman, J. W., Bauer, J. E., Waite, W. F., Osburn, C. L. & Chapman, N. R. Methane hydrate-bearing seeps as a source of aged dissolved organic carbon to the oceans. *Nat. Geosci.* **4**, 37–41 (2011).
9. Aleksandra, B. & Katarzyna, Ł.-M. Porewater dissolved organic and inorganic

carbon in relation to methane occurrence in sediments of the Gdańsk Basin (southern Baltic Sea). *Cont. Shelf Res.* **168**, 11–20 (2018).

10. Whiticar, M. J. Carbon and hydrogen isotope systematics of bacterial formation and oxidation of methane. *Chem. Geol.* **161**, 291–314 (1999).
