# Supplementary material for: Earthquake-enhanced dissolved carbon cycles in ultra-deep ocean sediments
Source: Nat Commun. 2023 Sep 11;14:5427. doi: 10.1038/s41467-023-41116-w (PMC10495447; doi:10.1038/s41467-023-41116-w)
Supplement: Supplementary file 3 — Reporting Summary [file 41467_2023_41116_MOESM3_ESM.pdf]

nature portfolio

Corresponding author(s): Rui Bao
Last updated by author(s): Aug 11, 2023

Reporting Summary

Nature Portfolio wishes to improve the reproducibility of the work that we publish. This form provides structure for consistency and transparency in reporting. For further information on Nature Portfolio policies, see our Editorial Policies and the Editorial Policy Checklist.

Statistics

For all statistical analyses, confirm that the following items are present in the figure legend, table legend, main text, or Methods section.

n/a	Confirmed	
☐	☒	The exact sample size (*n*) for each experimental group/condition, given as a discrete number and unit of measurement
☐	☒	A statement on whether measurements were taken from distinct samples or whether the same sample was measured repeatedly
☒	☐	The statistical test(s) used AND whether they are one- or two-sided<br>*Only common tests should be described solely by name; describe more complex techniques in the Methods section.*
☒	☐	A description of all covariates tested
☒	☐	A description of any assumptions or corrections, such as tests of normality and adjustment for multiple comparisons
☐	☒	A full description of the statistical parameters including central tendency (e.g. means) or other basic estimates (e.g. regression coefficient) AND variation (e.g. standard deviation) or associated estimates of uncertainty (e.g. confidence intervals)
☐	☒	For null hypothesis testing, the test statistic (e.g. *F*, *t*, *r*) with confidence intervals, effect sizes, degrees of freedom and *P* value noted<br>*Give P values as exact values whenever suitable.*
☒	☐	For Bayesian analysis, information on the choice of priors and Markov chain Monte Carlo settings
☒	☐	For hierarchical and complex designs, identification of the appropriate level for tests and full reporting of outcomes
☒	☐	Estimates of effect sizes (e.g. Cohen's *d*, Pearson's *r*), indicating how they were calculated

Our web collection on statistics for biologists contains articles on many of the points above.

Software and code

Policy information about availability of computer code

Data collection	Gebco2020 bathymetry data was used to generate Fig. 1a. IODP database (https://iodp.tamu.edu/) was used to generate Supplementary Fig. 6a.
Data analysis	Origin 2022 was used to analyze the data.

For manuscripts utilizing custom algorithms or software that are central to the research but not yet described in published literature, software must be made available to editors and reviewers. We strongly encourage code deposition in a community repository (e.g. GitHub). See the Nature Portfolio guidelines for submitting code & software for further information.

Data

Policy information about availability of data

All manuscripts must include a data availability statement. This statement should provide the following information, where applicable:

- Accession codes, unique identifiers, or web links for publicly available datasets
- A description of any restrictions on data availability
- For clinical datasets or third party data, please ensure that the statement adheres to our policy

Data used in this study are presented in Supplementary Information/Source Data file, and are also available on Figshare (doi: 10.6084/m9.figshare.23929950).

Research involving human participants, their data, or biological material

Policy information about studies with human participants or human data. See also policy information about sex, gender (identity/presentation), and sexual orientation and race, ethnicity and racism.

Reporting on sex and gender	n/a
Reporting on race, ethnicity, or other socially relevant groupings	n/a
Population characteristics	n/a
Recruitment	n/a
Ethics oversight	n/a

Note that full information on the approval of the study protocol must also be provided in the manuscript.

Field-specific reporting

Please select the one below that is the best fit for your research. If you are not sure, read the appropriate sections before making your selection.

☐ Life sciences ☐ Behavioural & social sciences ☒ Ecological, evolutionary & environmental sciences

For a reference copy of the document with all sections, see nature.com/documents/nr-reporting-summary-flat.pdf

Ecological, evolutionary & environmental sciences study design

All studies must disclose on these points even when the disclosure is negative.

Study description	This study reports concentrations, stable, and radiocarbon isotopic signatures of dissolved organic and inorganic carbon in the subsurface sediment interstitial water along the Japan Trench axis collected during the IODP Expedition 386.
Research sample	Samples used in this study were collected on R/V Kaimei in the Japan Trench during the IODP expedition 386 in 2021.
Sampling strategy	Samples were collected with up to 40 m long giant piston corers in the basins in the Japan Trench axis. Interstitial water samples were measured at resolution of one sample per 1 m.
Data collection	Geochemical analysis was carried out during the offshore phase of the IODP Expedition 386 following standard IODP procedure. Dissolved carbon samples were analyzed at the National Ocean Science Accelerator Mass Spectrometer (NOSAMS) facility at Woods Hole Oceanographic Institution for concentration, 13C and 14C analyses.
Timing and spatial scale	Interstitial water samples were collected on board R/V Kaimei immediately on core recovery during 13 April 2021 – 1 June 2021.
Data exclusions	No data were excluded.
Reproducibility	The residual samples are frozen and stored at Ocean University of China.
Randomization	No random grouping was used.
Blinding	No blinding was included.

Did the study involve field work? ☒ Yes ☐ No

Field work, collection and transport

Field conditions	Samples used in this study were collected in the Japan Trench axis.
Location	Summaries of IODP Holes used for geochemical analyses were reported as Supplementary Table 1.
Access & import/export	The International Ocean Discovery Program (IODP) Expedition 386: Japan Trench Paleoseismology was jointly funded by the European Consortium for Ocean Research Drilling (ECORD), the U.S. Science Support Program, and the IODP, with contributions and logistical support from the Japan Agency for Marine Earth Science and Technology (JAMSTEC).

Disturbance	Sediment sampling was restricted to methods and areas being essential to conduct the research goals.

Reporting for specific materials, systems and methods

We require information from authors about some types of materials, experimental systems and methods used in many studies. Here, indicate whether each material, system or method listed is relevant to your study. If you are not sure if a list item applies to your research, read the appropriate section before selecting a response.

Materials & experimental systems

n/a	Involved in the study
☒	☐ Antibodies
☒	☐ Eukaryotic cell lines
☒	☐ Palaeontology and archaeology
☒	☐ Animals and other organisms
☒	☐ Clinical data
☒	☐ Dual use research of concern
☒	☐ Plants

Methods

n/a	Involved in the study
☒	☐ ChIP-seq
☒	☐ Flow cytometry
☒	☐ MRI-based neuroimaging
